# Reference Intervals of Hematological Parameters Among Healthy Adults in Northern Sudan: A Community‐Based Cross‐Sectional Study

**DOI:** 10.1002/jcla.70061

**Published:** 2025-05-26

**Authors:** Mohamed F. Lutfi, Ahmed Ali Hassan, Ishag Adam

**Affiliations:** ^1^ Department of Physiology, College of Medicine Qassim University Buraidah Saudi Arabia; ^2^ Faculty of Medicine University of Khartoum Khartoum Sudan; ^3^ Department of Obstetrics and Gynecology, College of Medicine Qassim University Buraidah Saudi Arabia

**Keywords:** age, gender, hematological parameters, reference value

## Abstract

**Background:**

Recent studies from various countries, including those in Africa, have highlighted the importance of region‐specific data in assessing hematological health. However, no study has established hematological reference intervals (RIs) in Northern Sudan. We aimed to investigate the normal hematological values among apparently healthy adults in Northern Sudan.

**Methods:**

A cross‐sectional study was conducted in Northern Sudan. Standardized procedures measured participants' weight, height, and hematological parameters. The Mann–Whitney *U* test was used to compare the parameters between males and females. Mean, median, and RIs (2.5th–97.5th percentiles) were computed for the adults.

**Results:**

Two hundred fifty‐three adults were enrolled (141 [55.7%] males and 112 [44.3%] females). The median (interquartile [IQR]) of the enrolled adults' age was 40.0 (29.7–50.0) years. The RIs for both genders, hemoglobin 9.71–16.10 g/dL, red blood cells (RBC) 3.66–5.79 × 10^6^/mm^3^, hematocrit (HCT) 28.02%–46.75%, mean corpuscular volume (MCV) 64.02–95.65 fL, mean corpuscular hemoglobin (MCH) 21.57–33.80 pg, mean corpuscular hemoglobin concentration (MCHC) 31.60–36.56 g/dL, platelet count 155.35–454.30 × 10^3^/mm^3^, and total white blood cells (WBCs) 2.90–10.76 × 10^3^/mm^3^. Significantly higher median values were observed in males compared to females for hemoglobin, RBC, HCT, MCH, MCHC, and platelet distribution width. In contrast, females demonstrated significantly higher red cell distribution width‐coefficient of variation, WBC, platelet, mean platelet volume, and plateletcrit than males. MCV showed no significant difference between genders.

**Conclusion:**

The study's findings underscore the importance of establishing RIs for each region and specific gender population. RIs should also be established in other regions of Sudan to enhance clinical relevance and accuracy.

## Graphical abstract

Two hundred fifty‐three adults were enrolled in a cross‐sectional study conducted in Northern Sudan. The RVs of hemoglobin 9.71–16.10 g/dL, red blood cells (RBC) 3.66–5.79 × 10^6^/mm^3^, hematocrit (HCT) 28.02%–46.75%, mean corpuscular volume (MCV) 64.02–95.65 fL, mean corpuscular hemoglobin (MCH) 21.57–33.80 pg, mean corpuscular hemoglobin concentration (MCHC) 31.60–36.56 g/dL, platelet count 155.35–454.30 × 10^3^/mm^3^, and total white blood cells (WBCs) 2.90–10.76 × 10^3^/mm^3^. Significantly higher median values were observed in males compared to females for hemoglobin, RBC, HCT, MCH, MCHC, and platelet distribution width.

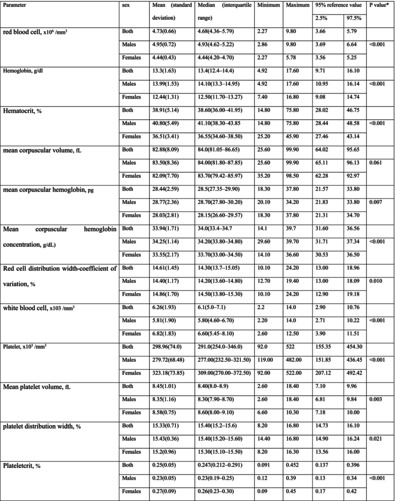

## 1 Introduction

Recent studies, including those from Africa, emphasize the need for region‐specific data in assessing hematological health [1, 2, 3, 4, 5]. Studying hematological reference intervals (RIs) among apparently healthy adults is essential for improving clinical diagnosis and management of hematological disorders in each region [1, 2, 3, 4, 5]. Those studies showed significant variations in hematological parameters, including hemoglobin, white blood cells (WBCs), and platelets, according to gender [1, 2, 3, 4, 5]. Hematological RIs are critical for interpreting laboratory results, guiding treatment decisions, and understanding population health trends. Such an approach is recommended, particularly in the context of limited resources in Africa.

Unlike Sudan, Ethiopia has established region‐specific hematological RIs, including in the Northeast [3], Northwest [6], East [2], and Southwest Ethiopia [1]. These studies also account for different age groups, such as children and adults [1, 2, 3] and geriatrics [7]. A recent study in India established the umbilical cord blood hematological parameters RI for newborns [8]. Establishing gender‐specific hematological RIs is crucial for accurate clinical diagnosis and effective patient management. Physiological differences between males and females, including hormonal influences, body composition, and metabolic variations, cause significant differences in the hematological parameters like hemoglobin, WBC counts, and platelet levels. These differences make it crucial to utilize gender‐specific RIs to avoid misinterpretations that could lead to inappropriate clinical decisions, particularly in populations with distinct health profiles and demographic characteristics [9, 10].

The RIs of hematological parameters have been studied among Sudanese adults in central [11, 12] and western regions [13]; significant differences were found between the areas and the genders. In addition, the RIs have been studied among different populations, including pregnant women [11] and children [12] in the central region. However, there is a noted scarcity of region‐specific hematological data, such as in Northern Sudan, which can lead to misinterpretations when using RIs derived from other populations. Northern Sudan's population has unique genetic and environmental factors, such as diet and altitude, that can influence hematological parameters. In Northern Sudan, the population faces unique health challenges influenced by factors such as nutritional status, dietary habits, prevalence of infectious diseases, and genetic diversity [14].

In Sudan, where standardized hematological RIs are lacking, current practice often relies on reference values derived from international guidelines. Some local laboratories and clinicians may also utilize limited, small‐scale studies conducted within Sudan for guidance, despite their potential limitations in generalizability. Such current practice in Sudan can lead to accurate diagnosis and monitoring challenges. For instance, women may experience lower hemoglobin levels due to menstrual blood loss and pregnancy, while men generally exhibit higher values. Therefore, having tailored RIs can enhance the detection of conditions such as anemia, leukopenia, or thrombocytopenia, which are critical for early intervention and treatment [9, 10]. Moreover, establishing gender‐specific RIs contributes to a better epidemiological understanding of hematological disorders, allowing for targeted public health interventions. By acknowledging and addressing gender differences in hematological values, healthcare providers can improve diagnostic accuracy and ultimately enhance patient outcomes. This approach is vital in diverse populations where cultural, nutritional, and environmental factors, such as those in Sudan, further influence health parameters. Furthermore, establishing hematological RIs in Northern Sudan will strengthen public health strategies, enhance diagnostic accuracy, and improve patient care in the region. Therefore, this study aimed to investigate the normal hematological values among apparently healthy adults in Almatamah, River Nile State, and Northern Sudan.

## 2 Materials and Methods

### 2.1 Study Area

River Nile State is located in northern Sudan. It is geographically situated between 16° and 22° north latitude and 32° and 35° east longitude. It is one of the 18 total states in Sudan. The total population of River Nile State was 1,240,440 persons [15]. There are seven localities in the River Nile State. Northern Sudan's distinct ethnic composition, centered on Arab and Nubian groups with an extended interaction history, its wheat‐based dietary traditions influenced by Nile agriculture and historical trade, and its generally lower altitude with an arid climate, all contribute to its unique region within Sudan. River Nile State exhibits distinct differences from the other areas of Sudan, particularly regarding ethnicity, dietary habits, and altitude. Most of the population of the River Nile State is descended from the Ja'alin tribes. These tribes have different cultures from those of other tribes in other regions of Sudan.

### 2.2 Study Population and Design

This community‐based cross‐sectional study was conducted from September to December 2022 at four villages in the Wad Hamid district (the lowest administrative unit in Sudan), Almatamah locality, River Nile State, Northern Sudan. The Wad Hamid district borders Khartoum State and is approximately 100 km from Khartoum city, the capital of Sudan.

Among the three districts of Almatamah locality, one was chosen randomly (Wad Hamid). Four villages were chosen from the randomly selected district using systematic sampling. Then, 50–70 households from each village were selected based on population density to obtain the desired sample size. Households were chosen at fixed intervals of every fifth household. The first member in each household who agreed to participate and met the study inclusion criteria was selected. If the house chosen was uninhabited or its inhabitants refused to participate, the next house was selected to meet the target number for the study. The investigators trained five medical officers in data collection methods to standardize the procedure and ensure high‐quality data.

After signing an informed consent form, all healthy adults (male and female) aged ≥ 18 were enrolled from the households using a lottery method. Participants whose age was less than 18 years, pregnant women who had donated or received blood within the past year, patients with poor cognitive functions (exhibiting clear signs of disorientation, inability to understand instructions, or provide coherent responses during the initial assessment), patients with chronic diseases such as diabetes mellitus, hypertension, hematological diseases, liver disease, recent infection, and severely ill patients were excluded from this study. Participants with diseases and/or conditions were excluded, as such diseases may influence the hematological values.

### 2.3 Data Collection

The authors followed Strengthening the Reporting of Observational Studies in Epidemiology (STROBE) guidelines to conduct this study [16]. The questionnaire was developed to collect the relevant data. The questionnaire included data on sociodemographic characteristics, such as age in years, sex (male or female), marital status (married or unmarried), educational level (< secondary or ≥ secondary), and occupational status (employed or unemployed). Anthropometric measurements were obtained, including weight and height (the latter expressed as body mass index [BMI]). Blood samples were obtained from all participants for complete blood count (CBC) profile (hematological parameters) including hemoglobin, RBC, WBC, platelets, red blood cell (RBC), hematocrit (HCT), mean corpuscular volume (MCV), mean corpuscular hemoglobin (MCH), mean corpuscular hemoglobin concentration (MCHC), red cell distribution width‐coefficient of variation (RDW‐CV), mean platelet volume (MPV), platelet distribution width (PDW), and plateletcrit (PCT).

### 2.4 Anthropometric Measurements

Each participant's weight was measured in kilograms using well‐calibrated scales and adjusted to zero before each measurement as the standard procedure. The participant stood with minimal movement, with hands by their sides and shoes and excess clothing removed. The participant's height was measured in centimeters after standing straight with their back against a wall and feet together. BMI was computed as the weight in kilograms divided by the square of the height in meters (kg/m^2^) [17].

### 2.5 Blood Analysis

Each participant was requested to provide 3 mL of blood drawn in an ethylenediaminetetraacetic acid tube under aseptic conditions. Blood samples were transported in a cool box with ice packs to maintain a temperature between 2°C and 8°C and analyzed within 6 h to minimize cellular degradation and maintain accurate hematological parameters. These samples were used to perform the CBC parameters as per the manufacturer's instructions (Sysmex KX‐21, Japan). Analyzer validation and calibration were done to ensure the performance of hematology analyzer according to the manufacturer's specifications and is fit for clinical use before it is used for patient testing. In addition, all internal quality controls and external validation procedures were followed.

### 2.6 Ethics Approval and Consent to Participate

The current work was conducted according to the Declaration of Helsinki. Almatamah Health Authority, Sudan, approved this study. The reference is #9, 2021. All participants signed the written informed consent. The authors followed all measures to ensure participants' privacy, confidentiality, and safety, such as excluding personal identifiers during data collection.

### 2.7 Sample Size Calculation

As per the recommendation from the Clinical and Laboratory Standards Institute (CLSI), a minimum of 120 individuals is sufficient to establish RIs [18, 19]. The authors increased the sample size to 253 to enhance the reliability of the results. While CLSI suggests 120 individuals, our larger sample size of 253 was chosen to enhance the statistical power and robustness of our RIs estimations, particularly for capturing the variability within our specific Northern Sudanese population. This increased sample aims to provide more precise and reliable reference values for clinical application in the region.

### 2.8 Statistical Analysis

The IBM Statistical Package analyzed the Social Sciences (SPSS) data for Windows, version 22.0 (SPSS Inc., New York, USA). The proportions were expressed as percentages (%). Continuous data such as age, BMI, and hematological parameters were evaluated for normality using the Kolmogorov–Smirnov test, and they were found to be non‐normally distributed. Therefore, they were expressed using the median (interquartile range [IQR]). In addition, besides median (IQR), hematological parameters were described by the mean (SD). The Mann–Whitney *U* test was used to compare medians between genders and the different tested hematological parameters. The 95% (2.5th and 97.5th percentiles) and the minimum and maximum limits for the hematological parameters were computed. A two‐sided *p*‐value of < 0.05 was considered statistically significant.

## 3 Results

### 3.1 The Study Participants' Characteristics

Two hundred fifty‐three adults were enrolled; 141 (55.7%) were males and 112 (44.3%) were females. The median (IQR) of the enrolled adults of the age and BMI was 40.0 (29.7–50.0) years and 25.15 (21.68–30.31) kg/m^2^, respectively. Of the 253 adults, 166 (65.6%) and 87(34.4%) had education ≥ secondary and < secondary level, respectively. Seventy‐seven (30.4%) of the adults were married. One hundred twenty‐eight (50.6%) of the adults were employed (Table 1).

**TABLE 1:** Characteristics of the studied healthy adults in Northern Sudan, 2022 (*n* = 253).

Variables	Median	Interquartile range
Age, years	40.0	29.7–50.0
Body mass index, kg/m^2^	25.15	21.68–30.31

	Number	%
Sex		
Male	141	55.7
Female	112	44.3
Education		
≥ Secondary	166	65.6
< Secondary	87	34.4
Occupation status		
Employed	128	50.6
Unemployed	125	49.4
Marital status		
Married	77	30.4
Unmarried	176	69.6

### 3.2 Descriptive Statistic of the Tested Hematological Parameters

Table 2 shows the descriptive statistics of the tested hematological parameters, including the mean (SD), median (IQR), minimum, maximum, and 95% RI (2.5% and 97.5%). The RIs of hematological parameters showed hemoglobin 9.71–16.10 g/dL, RBC 3.66–5.79 (×10^6^/mm^3^), HCT 28.02%–46.75%, MCV 64.02–95.65 fL, MCH 21.57–33.80 pg, MCHC 31.60–36.56 g/dL, platelet 155.35–454.30 × 10^3^/mm^3^, and WBC 2.90–10.76 × 10^3^/mm^3^.

**TABLE 2:** Descriptive statistics and 95% reference values of hematological parameters in relation to sex (141 males and 112 females) for healthy adults in Northern Sudan (*N* = 253).

Parameter	Sex	Mean (standard deviation)	Median (interquartile range)	Minimum	Maximum	95% Reference value		*p* ^a^
						2.5%	97.5%	
Red blood cell, ×10^6^/mm^3^	Both	4.73 (0.66)	4.68 (4.36–5.79)	2.27	9.80	3.66	5.79	
	Males	4.95 (0.72)	4.93 (4.62–5.22)	2.86	9.80	3.69	6.64	< 0.001
	Females	4.44 (0.43)	4.44 (4.20–4.70)	2.27	5.78	3.56	5.25	
Hemoglobin, g/dL	Both	13.3 (1.63)	13.4 (12.4–14.4)	4.92	17.60	9.71	16.10	
	Males	13.99 (1.53)	14.10 (13.3–14.95)	4.92	17.60	10.95	16.14	< 0.001
	Females	12.44 (1.31)	12.50 (11.70–13.27)	7.40	16.80	9.08	14.74	
Hematocrit, %	Both	38.91 (5.14)	38.60 (36.00–41.95)	14.80	75.80	28.02	46.75	
	Males	40.80 (5.49)	41.10 (38.30–43.85)	14.80	75.80	28.44	48.58	< 0.001
	Females	36.51 (3.41)	36.55 (34.60–38.50)	25.20	45.90	27.46	43.14	
Mean corpuscular volume, fL	Both	82.88 (8.09)	84.0 (81.05–86.65)	25.60	99.90	64.02	95.65	
	Males	83.50 (8.36)	84.00 (81.80–87.85)	25.60	99.90	65.11	96.13	0.061
	Females	82.09 (7.70)	83.70 (79.42–85.97)	35.20	98.50	62.28	92.97	
Mean corpuscular hemoglobin, pg	Both	28.44 (2.59)	28.5 (27.35–29.90)	18.30	37.80	21.57	33.80	
	Males	28.77 (2.36)	28.70 (27.80–30.20)	20.10	34.20	21.83	33.80	0.007
	Females	28.03 (2.81)	28.15 (26.60–29.57)	18.30	37.80	21.31	34.70	
Mean corpuscular hemoglobin concentration, g/dL	Both	33.94 (1.71)	34.0 (33.4–34.7)	14.1	39.7	31.60	36.56	
	Males	34.25 (1.14)	34.20 (33.80–34.80)	29.60	39.70	31.71	37.34	< 0.001
	Females	33.55 (2.17)	33.70 (33.00–34.50)	14.10	36.60	30.53	36.50	
Red cell distribution width‐coefficient of variation, %	Both	14.61 (1.45)	14.30 (13.7–15.05)	10.10	24.20	13.00	18.96	
	Males	14.40 (1.17)	14.20 (13.60–14.80)	12.70	19.40	13.00	18.09	0.010
	Females	14.86 (1.70)	14.50 (13.80–15.30)	10.10	24.20	12.90	19.18	
White blood cell, ×10^3^/mm^3^	Both	6.26 (1.93)	6.1 (5.0–7.1)	2.2	14.0	2.90	10.76	
	Males	5.81 (1.90)	5.80 (4.60–6.70)	2.20	14.0	2.71	10.22	< 0.001
	Females	6.82 (1.83)	6.60 (5.45–8.10)	2.60	12.50	3.90	11.51	
Platelet, ×10^3^/mm^3^	Both	298.96 (74.0)	291.0 (254.0–346.0)	92.0	522	155.35	454.30	
	Males	279.72 (68.48)	277.00 (232.50–321.50)	119.00	482.00	151.85	436.45	< 0.001
	Females	323.18 (73.85)	309.00 (270.00–372.50)	92.00	522.00	207.12	492.42	
Mean platelet volume, fL	Both	8.45 (1.01)	8.40 (8.0–8.9)	2.60	18.40	7.10	9.96	
	Males	8.35 (1.16)	8.30 (7.90–8.70)	2.60	18.40	6.81	9.84	0.003
	Females	8.58 (0.75)	8.60 (8.00–9.10)	6.60	10.30	7.18	10.00	
Platelet distribution width, %	Both	15.33 (0.71)	15.40 (15.2–15.6)	8.20	16.80	14.73	16.10	
	Males	15.43 (0.36)	15.40 (15.20–15.60)	14.40	16.80	14.90	16.24	0.021
	Females	15.2 (0.96)	15.30 (15.10–15.50)	8.20	16.30	13.56	16.00	
Plateletcrit, %	Both	0.25 (0.05)	0.247 (0.212–0.291)	0.091	0.452	0.137	0.396	
	Males	0.23 (0.05)	0.23 (0.19–0.25)	0.12	0.39	0.13	0.34	< 0.001
	Females	0.27 (0.09)	0.26 (0.23–0.30)	0.09	0.45	0.17	0.42	

^a^ Comparison of medians between genders using Mann–Whitney *U* test.

Significantly higher median values were observed in males compared to females for hemoglobin (14.10 vs. 12.50 g/dL, *p* < 0.001), RBC (4.93 vs. 4.44 × 10^6^/mm^3^, *p* < 0.001), HCT (41.10% vs. 36.55%, *p* < 0.001), MCH (28.70 vs. 28.15 pg, *p* = 0.007), MCHC (34.20 vs. 33.70 g/dL, *p* < 0.001), and PDW (15.40% vs. 15.30%, *p* = 0.021). In contrast, females demonstrated significantly higher RDW‐CV (14.50% vs. 14.20%, *p* = 0.010), WBC (6.60 vs. 5.80 × 10^3^/mm^3^, *p* < 0.001), platelet (309.0 vs. 277.0 × 10^3^/mm^3^, *p* < 0.001), MPV (8.60 vs. 8.30 fL, *p* = 0.021), and PCT (0.26% vs. 0.23%, *p* < 0.001) compared to males. MCV showed no significant difference between genders (Table 2).

## 4 Discussion

The main findings of this study were that almost all hematological parameters, apart from MCV, significantly differed between both genders (males vs. females), and it provided RIs that can be used in Northern Sudan. As earlier mentioned, no RIs for hematological parameters exist for adults in the north of Sudan compared to the western and central regions of Sudan [11, 12, 13]. Even those studies conducted in other areas of Sudan have typically investigated a more limited panel of hematological parameters compared to the comprehensive assessment undertaken in this study. Table 3 compares the RIs obtained in Northern Sudan with the findings of other studies conducted in different regions of Sudan. Compared to studies from Central [11, 12] and Western [13] Sudan, this cohort exhibited relatively lower RBC count, hemoglobin, HCT, and MCV values, while other RBC indices showed varying trends. These disparities may reflect regional differences in altitude, nutrition, or endemic diseases. For example, in central Sudan, Khartoum's urban population is more likely to benefit from diverse diets and better healthcare access. In contrast, Northern Sudan's rural communities face limited iron‐rich food availability and higher malaria prevalence, suppressing erythropoiesis [20, 21]. For example, dietary surveys in the Almatamah Locality indicate low consumption of meat and legumes, key iron sources, compared to urban centers [22]. Chronic infections like soil‐transmitted helminths, prevalent in the River Nile State, may exacerbate anemia, further lowering hemoglobin and HCT levels [23].

**TABLE 3:** Comparison of the key hematological parameters among healthy adults in this study and other studies in Sudan.

Parameter	Sex	This study			Western Sudan [13]		Central Sudan [11, 12]	
		Mean	Median	Reference interval (RI)	Mean	RI	Mean	RI
Red blood cell, ×10^6^/mm^3^	Both	4.73	4.68	3.66–5.79	/	/	/	/
	Males	4.95	4.93	3.69–6.64	5.48	4.6–6.3	5.2	4.7–5.7
	Females	4.44	4.44	3.56–5.25	4.88	4.1–5.6	4.5	4.1–4.9
Hemoglobin, g/dL	Both	13.3	13.4	9.71–16.10	/	/	/	/
	Males	13.99	14.10	10.95–16.14	15.7	12.7–18.6	14.9	13.4–16.4
	Females	12.44	12.50	9.08–14.74	13.38	10.4–16.2	12.2	10.7–13.7
Hematocrit, %	Both	38.91	38.60	28.02–46.75	/	/	/	/
	Males	40.80	41.1	28.44–48.58	49.73	41.8–57.5	46.4	41.1–51.7
	Females	36.51	36.55	27.46–43.14	43.54	32.3–54.7	39.2	34.2–44.2
Mean corpuscular volume, fL	Both	82.88	84.0	64.02–95.65	/	/	/	/
	Males	83.5	84.0	65.11–96.13	91.34	81.6–101.0	89.8	80.5–99.1
	Females	82.09	83.7	62.28–92.97	89.24	75.9–102.4	86.2	76.4–96
Mean corpuscular hemoglobin, pg	Both	28.44	28.5	21.57–33.80	/	/	/	/
	Males	28.77	28.7	21.83–33.80	28.73	24.4–33.0	28.7	25.9–31.5
	Females	28.03	28.15	21.31–34.70	27.45	21.9–32.9	27.3	23.3–31.3
Mean corpuscular hemoglobin concentration, g/dL	Both	33.94	34.0	31.60–36.56	/	/	/	/
	Males	34.25	34.20	31.71–37.34	31.47	29.6–33.0	32.2	29.5–34.9
	Females	33.55	33.7	30.53–36.50	30.74	28.7–32.7	31.4	28.9–33.9
Red cell distribution width‐coefficient of variation, %	Both	14.61	14.30	13.00–18.96	/	/	/	/
	Males	14.4	14.2	13.00–18.09	13.8	11.9–15.6	/	/
	Females	14.86	14.50	12.90–19.18	14.84	9.5–20.0	/	/
White blood cell, ×10^3^/mm^3^	Both	6.26	6.1	2.90–10.76	/	/	5.01	/
	Males	5.81	5.80	2.71–10.22	5.28	2.5–8.0	4.96	/
	Females	6.82	6.60	3.90–11.51	6.01	2.8–9.1	5.13	/
Platelet, ×10^3^/mm^3^	Both	298.96	291.0	155.35–454.30	/	/	/	/
	Males	279.72	277.0	151.85–436.45	232.43	85.4–379.0	/	/
	Females	323.18	309.0	207.12–492.42	312.64	139.7–485.5	/	/
Mean platelet volume, fL	Both	8.45	8.40	7.10–9.96	/	/	/	/
	Males	8.35	8.30	6.81–9.84	8.74	7.6–9.8	/	/
	Females	8.58	8.60	7.18–10.00	8.78	7.4–10.0	/	/
Platelet distribution width, %	Both	15.33	15.40	14.73–16.10	/	/	/	/
	Males	15.43	15.40	14.90–16.24	15.8	14.9–16.6	/	/
	Females	15.2	15.30	13.56–16.00	15.53	14.8–16.2	/	/
Plateletcrit, %	Both	0.25	0.247	0.137–0.396	/	/	/	/
	Males	0.23	0.23	0.13–0.34	/	/	/	/
	Females	0.27	0.26	0.17–0.42	/	/	/	/

Platelet counts in Northern Sudan aligned closely with Western Sudan [13], probably because of shared environmental/genetic factors. However, WBC counts were lower than those in Central Sudan, possibly due to reduced exposure to urban pathogens and pollutants or genetic adaptations to endemic infections [23]. Based on the discussion above, it is evident that RIs' regional heterogeneity precludes their use as national RIs for all Sudanese people and highlights the urgent need for region‐specific hematological benchmarks tailored to Sudan's ecological and epidemiological diversity.

Table 4 comprehensively compares hematological parameters obtained from this study with those reported in comparable studies from nearby countries, including Eritrea [5], Ethiopia [1], Mali [24], Zimbabwe [25], and Yemen [4]. An in‐depth analysis of these RIs reveals significant variability across populations, which can be explained by concurrent differences in genetic, environmental, nutritional, and socioeconomic factors in these countries [9, 20, 21, 22, 23, 26, 27, 28].

**TABLE 4:** Comparison of the key hematological parameters among healthy adults between this study and some countries.

Parameter	Sex	This study			Ethiopia [1]		Eritrea [5]			Mali [24]		Zimbabwe [25]			Yemen [4]		
		Mean	Median	Reference interval (RI)	Median	RI	Mean	Median	RI	Median	RI	Mean	Median	RI	Mean	Median	RI
Red blood cell, ×10^6^/mm^3^	Both	4.73	4.68	3.66–5.79	/	/	5.18	5.06	4.02–7.19	/	/	5.2	5.1	4.1–6.6	4.8	4.8	3.9–6.3
	Males	4.95	4.93	3.69–6.64	5.32	4.26–6.68	5.57	5.45	4.47–7.69	5.1	4.1–6.2	5.5	5.5	4.4–6.7	5.1	5.1	4.1–6.2
	Females	4.44	4.44	3.56–5.25	5.02	4.02–6.16	4.97	4.90	3.98–6.38	4.7	3.9–5.7	4.8	4.7	3.9–5.9	4.4	4.4	3.8–5.5
Hemoglobin, g/dL	Both	13.3	13.4	9.71–16.10	/	/	14.13	13.98	11.03–17.62	/	/	14.7	14.8	10.5–18.0	14.33	14.30	11.16–17.54
	Males	13.99	14.10	10.95–16.14	15.5	12.1–18.8	15.28	15.42	11.48–17.99	14.5	12.4–17.6	15.8	15.9	13.2–18.3	15.31	15.30	12.73–17.60
	Females	12.44	12.50	9.08–14.74	14.6	12.3–17.9	13.50	13.49	10.74–16.54	12.8	12.0–14.9	13.4	13.5	10.2–15.9	13.06	13.0	10.65–15.35
Hematocrit, %	Both	38.91	38.60	28.02–46.75	/	/	45.15	44.4	34.86–58.60	/	/	45.3	45.4	34.7–54.2	40.9	40.8	33.03–49.3
	Males	40.80	41.1	28.44–48.58	45.2	36.72–54.48	48.75	48.5	38.96–61.17	/	/	48.5	48.5	42.0–55.1	43.45	43.6	36.54–49.3
	Females	36.51	36.55	27.46–43.14	43.1	36.86–51.59	43.19	42.9	34.36–54.74	/	/	41.7	41.7	33.9–48.7	37.4	37.4	31.8–43.44
Mean corpuscular volume, fL	Both	82.88	84.0	64.02–95.65	/	/	87.67	88.0	76.56–97.29	/	/	88.1	88.5	70.8–102.2	85.73	86.2	72.83–94.55
	Males	83.5	84.0	65.11–96.13	84.3	74.8–93.94	88.04	88.60	76.72–98.57	/	/	88.7	88.8	72.8–102.6	86.82	86.3	72.48–94.38
	Females	82.09	83.7	62.28–92.97	86.2	77.3–98.82	87.46	87.7	76.5–96.68	/	/	87.4	88.2	68.8–100.7	85.53	86.0	72.33–94.81
Mean corpuscular hemoglobin, pg	Both	28.44	28.5	21.57–33.80	/	/	27.53	28.3	20.46–32.70	/	/	28.5	28.9	21.9–32.9	30.06	30.4	23.95–33.55
	Males	28.77	28.7	21.83–33.80	29.0	24.86–32.84	27.83	28.8	19.90–33.60	/	/	29.0	29.4	22.9–33.5	30.24	30.4	23.85–33.85
	Females	28.03	28.15	21.31–34.70	29.4	26.3–33.58	27.37	28.1	20.79–31.30	/	/	28.0	28.4	20.7–32.1	29.84	30.1	24.02–33.50
Mean corpuscular hemoglobin concentration, g/dL	Both	33.94	34.0	31.60–36.56	/	/	31.38	32.2	25.20–35.30	/	/	32.4	32.4	29.5–35.0	35.02	35.1	32.97–36.73
	Males	34.25	34.20	31.71–37.34	343.0	320.6–365.0	31.55	32.4	25.30–35.9	/	/	32.7	32.7	29.8–35.4	35.18	35.3	32.93–36.77
	Females	33.55	33.7	30.53–36.50	339.5	320.0–360.0	31.82	32.1	25.10–33.50	/	/	32.0	32.1	29.2–34.3	34.83	34.9	32.94–36.76
Red cell distribution width‐coefficient of variation, %	Both	14.61	14.30	13.00–18.96	/	/	14.65	14.2	12.70–18.60	/	/	/	/	/	/	/	/
	Males	14.4	14.2	13.00–18.09	13.7	12.46–17.56	14.96	14.1	12.70–19.58	/	/	/	/	/	/	/	/
	Females	14.86	14.50	12.90–19.18	13.6	12.4–15.59	14.62	14.3	12.63–18.57	/	/	/	/	/	/	/	/
White blood cell, ×10^3^/mm^3^	Both	6.26	6.1	2.90–10.76	/	/	6.83	6.37	3.02–13.55	/	/	5.1	4.9	2.9–7.9	5.25	5.1	2.810–8.80
	Males	5.81	5.80	2.71–10.22	6.36	3.31–11.62	6.63	6.03	2.47–14.18	5.2	3.07–11.1	4.85	4.6	2.8–8.1	5.23	4.97	2.85–8.94
	Females	6.82	6.60	3.90–11.51	6.34	3.24–10.05	6.93	6.47	3.35–13.29	4.7	3.8–12.5	5.4	5.2	3.3–8.3	5.26	5.20	2.75–8.46
Platelet, ×10^3^/mm^3^	Both	298.96	291.0	155.35–454.30	/	/	286.8	281.2	131.62–453.13	/	/	252.2	247.0	138–384.8	270.4	269.0	140.0–418.6
	Males	279.72	277.0	151.85–436.45	275.0	164.0–403.4	277.2	274.3	151.05–405.7	259.0	133–460	231.2	229.0	125.3–257.0	264.1	261.0	147.7–411.2
	Females	323.18	309.0	207.12–492.42	288.0	202.3–444.5	292.02	285.1	117.8–467.38	291.0	151–460	276.3	268.5	163.8–431.0	278.2	278.5	140.3–424.1
Mean platelet volume, fL	Both	8.45	8.40	7.10–9.96	/	/	8.92	8.84	7.28–11.01	/	/	10.3	10.2	8.7–12.3	/	/	/
	Males	8.35	8.30	6.81–9.84	/	/	8.69	8.66	7.03–10.66	/	/	10.3	10.2	8.7–12.4	/	/	/
	Females	8.58	8.60	7.18–10.00	/	/	9.04	8.95	7.48–11.17	/	/	10.3	10.2	8.7–12.3	/	/	/
Platelet distribution width, %	Both	15.33	15.40	14.73–16.10	/	/	/	/	/	/	/	/	/	/	/	/	/
	Males	15.43	15.40	14.90–16.24	/	/	/	/	/	/	/	/	/	/	/	/	/
	Females	15.2	15.30	13.56–16.00	/	/	/	/	/	/	/	/	/	/	/	/	/
Plateletcrit, %	Both	0.25	0.247	0.137–0.396	/	/	/	/	/	/	/	/	/	/	/	/	/
	Males	0.23	0.23	0.13–0.34	/	/	/	/	/	/	/	/	/	/	/	/	/
	Females	0.27	0.26	0.17–0.42	/	/	/	/	/	/	/	/	/	/	/	/	/

In the present study, hemoglobin RIs were lower than those reported in countries such as Eritrea [5] and Ethiopia [1]. HCT values for both sexes in Northern Sudan were notably lower compared to levels observed in Eritrea [5] and Mali [24]. Likewise, the RBC count was also reduced relative to values from Eritrea [5] and Yemen [4]. Higher RBC indices in Eritrea [5], Ethiopia [1], and Yemen [4] are secondary to high altitude, which enhances the process of erythropoiesis [29]. In addition, the lower hemoglobin and HCT levels in Northern Sudan may be attributed to limited access to iron‐rich diets, chronic malnutrition, parasitic infections, such as malaria and hookworm infestations, poor healthcare infrastructure, and low altitude [20, 21].

The MCV values in Northern Sudan overlapped with those in Eritrea [5] but were broader than those in Ethiopia [1]. The wider range suggests more significant heterogeneity in RBC size, possibly due to mixed nutritional deficiencies or varying degrees of anemia severity. The MCH and MCHC RIs in Northern Sudan were comparable to those in Eritrea and Ethiopia, indicating that the hemoglobin content per cell was relatively consistent across these populations. However, the RDW‐CV values in Northern Sudan were slightly higher than those in Eritrea [5], suggesting more significant variability in RBC size [30].

In Northern Sudan, the WBC RIs were generally lower than those in Eritrea [5] and Mali [24] but higher than those in Zimbabwe [25] and Yemen [4]. These differences may reflect variations in endemic infectious diseases because populations exposed to frequent infections often exhibit higher baseline WBC counts. Moreover, there is a relatively decreased upper limit of WBC RIs in Northern Sudan than in Eritrea [5] and Mali [24], which indicates better control of infectious diseases or differences in population health status [30]. On the other hand, the lower limit of WBC RIs in Northern Sudan was similar to those in different countries, suggesting that severe leukopenia is rare across these populations [30]. Further research is needed to explore the impact of chronic infections, such as malaria and tuberculosis, on WBC parameters in Northern Sudan.

The platelet count RIs in Northern Sudan were higher than those in Zimbabwe [25] and Yemen [4]. The MPV RIs were slightly lower than those in Eritrea [5]. Notably, the PDW and PCT RIs were unavailable for most countries, making direct comparisons with those from Northern Sudan challenging. The higher platelet counts and slightly lower MPV in Northern Sudan compared to the regions above may result from a complex interplay of genetic predispositions and environmental influences [23]. Further studies must elucidate the factors contributing to these platelet indices' differences.

The observed gender disparities of the studied hematological RIs align with global trends; however, they highlight some differences that may be specific to Northern Sudan. Males exhibited significantly higher median values for hemoglobin, RBC count, and hematocrit than females. Physiologically, greater muscle mass in males increases oxygen demand and, consequently, erythropoiesis [31]. There is repeated evidence that testosterone enhances erythropoietin sensitivity [32] while estrogens suppress hepcidin, modulate iron availability, and therefore contribute to lower RBC indices in females [33]. The later hypophysis also explains significantly higher RDW‐CV in the studies of females [34]. Regular menstrual cycles can lead to periodic blood loss, causing increased RBC turnover and more variation in RBC size. This can lead to a higher proportion of reticulocytes, which vary in size, increasing RDW‐CV [34]. Recognizing that menstruation can affect hematological profiles, future research should aim to determine separate reference intervals for menstruating and non‐menstruating women, as this study did not exclude participants based on their menstrual cycle.

In contrast, some researchers believed that the observed difference in RBC indices does not necessarily correlate with variations in iron status or erythropoietin levels between males and females [35]. Instead, renal rather than bone marrow mechanisms may explain this phenomenon. Female estrogens promote vasodilatation, while male androgens induce vasoconstriction of the renal arterioles [35]. In blood vessels smaller than 300 μm, vasodilatation raises hematocrit levels, whereas vasoconstriction does the opposite. This mechanism enables fine‐tuning oxygen delivery without increasing or decreasing erythropoiesis [35].

Although MCV showed no significant gender difference in the present study, MCH and MCHC were significantly higher in males than in females. This suggests that male androgens may significantly influence hemoglobin synthesis more than RBC size; however, this hypothesis remains to be explored by further research.

The present study also demonstrated significantly higher WBCs, platelet count, MPV, and PCT in females than in males. Females tend to have higher baseline inflammatory markers due to hormonal fluctuations, particularly during the menstrual cycle [36]. This can result in a slightly elevated WBC count compared to males [37]. Likewise, periodic bleeding during menstruation necessitates efficient hemostatic control in females. This is partly fulfilled by female sex hormones, which stimulate megakaryopoiesis, leading to a higher platelet count in females [37]. Physiologically, activated platelets become swollen, leading to increased MPV. Higher MPV in females suggests increased platelet activation, which can occur under the effects of estrogens and progesterone [9]. Since both platelet count and MPV are higher in females, PCT naturally increases because, mathematically, PCT = platelet count × MPV/10. Likewise, higher platelet count, turnover, and activation decrease the variability in platelet size and consequently, PDW [38]. In conclusion, it seems evident that high platelet count, MPV, PCT, and low PDW in females are among the protective mechanisms that ensure adequate hemostasis and prevent excessive menstrual bleeding during reproductive years [9].

These examples show the potential pathophysiological significance of abnormal values for key hematological parameters. For instance, deviations in hemoglobin can indicate anemia or polycythemia with associated oxygen transport implications, while abnormal WBCs may suggest infection or hematological disorders [39].

## 5 Limitations and Recommendations

While this study adhered to CLSI guidelines and strengthened validity with a sample size exceeding the recommendation, limitations must be acknowledged. First, the restriction to the Wad Hamid district limits generalizability to all Northern Sudanese populations. Future studies should incorporate multi‐center designs across Sudan's 18 states to capture broader ethnic and environmental diversity. Second, the exclusion of vulnerable groups like pregnant women, children, and the elderly overlooks critical demographics. Third, the cross‐sectional design cannot account for seasonal variations in some hematological parameters like WBC counts, which may fluctuate with malaria transmission peaks [21]. Finally, another limitation of this study is the absence of routine urine and stool examinations for the participants. While detailed questioning about current illnesses and observed health abnormalities served as an exclusion criterion, subclinical infections or asymptomatic conditions detectable through laboratory analysis may have been present and could potentially influence the hematological RIs established.

This study underscores the critical need for standardized national hematological RIs in Sudan, emphasizing the importance of establishing region‐ and gender‐specific RIs. The absence of such standardized RIs currently limits diagnostic accuracy and optimal patient management nationwide. Future research must integrate genetic testing (e.g., hemoglobin electrophoresis) and nutritional biomarkers (e.g., serum ferritin) to exclude possible genetic, dietary, and infectious causes of the observed variations in the studied hematological RIs. Likewise, longitudinal studies tracking hematological changes across seasons or life stages would further refine RIs. Also, future research should consider the potential influence of methodological variations across studies on hematological profiles and the impact of chronic infections and genetic adaptations prevalent in the population on hematological profiles to establish more context‐specific reference intervals. Collaboration with Sudan's Federal Ministry of Health to consider these RIs in national guidelines could standardize laboratory practices and improve patient management.

## 6 Conclusion

The study's findings underscore the importance of establishing RIs tailored to each region and specific population by gender, as advocated by researchers. It successfully established region‐specific hematological RIs per gender for Northern Sudan. To enhance clinical relevance and accuracy, establishing RIs in other regions of Sudan, such as the Eastern region, is essential.

## Author Contributions

M.F.L. and I.A. conceived the study; A.A.H. and I.A. supervised the work, guided the analysis, and critically reviewed the manuscript; M.F.L. and I.A. prepared the analysis plan, performed the data analysis, wrote the first draft of the paper, and supervised data collection. All authors reviewed and approved the final manuscript.

## Consent

The authors have nothing to report.

## Conflicts of Interest

The authors declare no conflicts of interest.

## Data Availability

The data are available from the corresponding author upon reasonable request.
